# Books and Authors

**Published:** 1909-03

**Authors:** 


### BOOKS AND AUTHORS.

---

**The Surgery of the Ear.** By **Samuel J. Kopetzky,** M. D., Attending Otologist, New York City Children's Hospitals and Schools. Octavo, pp. 385. Illustrated. New York: Rebman Co. (Cloth, $4.00.)

A few years ago one would be startled almost to see a book with this title, a work limited to the surgery of the ear,—to one organ of special sense. And yet from the present outlook it is more than likely that, in the near future, we will observe men devoting their entire time to the treatment of the diseases of the ear. The advances of medical knowledge pertaining to the ear have been such, of late, as to justify the issuance of such a treatise as this, and Kopetzky is well qualified to undertake the task.

The radical mastoid operation, now attracting so much attention, receives elaborate treatment at this author's hands. He discusses all the various flaps and procedures, and illustrates the chapter admirably. He deals with the minor diseases and procedures as well as the major and thus makes a book equally valuable for the student, general practitioner, and specialist. It is a work of art, mechanically speaking, reflecting credit alike upon author, artist, and publisher.

**Principles and Practice of Physical Diagnosis.** By **John C. DaCosta,** Jr., Assistant in Clinical Medicine in Jefferson Medical College, Philadelphia. Octavo, pp. 548. Illustrated. Philadelphia and London: W. B. Saunders Co. (Cloth, $3.50.)

Following in the footsteps of his illustrious father, the younger DaCosta has created a work on physical diagnosis that might well challenge the admiration of the senior were he present to inspect it. One of the special features of this book is its size. It is sufficiently comprehensive for practical use, while it remains within the limits of convenience. The author has taught at the bedside for ten years and has accumulated a wealth of material from two large hospitals, that he has utilised in the preparation of his book. The illustrations are such as to make the text speak with forcefulness, accentuating every point of significance. Many of the pictures are from living models, either sick or well, according to the condition or fact being developed.

The text shows the handiwork of a scholar, the rhetoric is choice, and there is no repetition. Diphthongs are eliminated and compound words are few, making the page attractive to the trained eye.

DaCosta, it is almost needless to say, is strong in pulmonary and cardiovascular diagnosis; indeed, these regions are always the favorites in teaching physical diagnosis. On the other hand he does not neglect any region or locality that can be demonstrated in such a work. It may be stated, as a conclusion, that this is one of the very useful books of the period, and will surely be adopted by the larger schools of medicine as a textbook on physical diagnosis.

---

**On the Means of the Prolongation of Life,** by **Sir Hermann Weber,** M.D., F.R., C.P. Consulting Physician to the German Hospital, the National Hospital for consumption, Ventnor, the Mount Vernon Hospital for consumption and a member of the consulting committee of King Edward VII sanatorium at Midhurst, London. John Bale, Sons, and Danielsson, L't'd., Oxford House 83-91 Great Titchfield street, Oxford street, West. 1908. (Price, $1.25 net.)

It is a genuine pleasure to read such a book as this,—to read this book,—so full of intellectual thought on a most important topic. If every person whose life is worth preserving should follow Sir Hermann Weber's advice regarding methods and habits of life, and especially with regard to eating and drinking, it would be safe to predict an increase in the average length of years of human life, and thus the diminution of mortality tables of the aged.

The importance of exercise is pretty generally understood by intelligent persons, but it is so effectively portrayed here as to make its results on the various organs more clearly understood. Exercise need not be violent, but it should be regular and of the kind adapted to the individual, sometimes more prolonged, again milder and of shorter duration. Respiratory exercises especially are to be commended, and when, for any reason they cannot be taken actively, as in walking or climbing, then they should be taken passively by slowly and rhythmically expanding and contracting the chest. Exercise of the muscles is of great benefit to a weak heart, and muscle tension should be practised by such persons. Sir Hermann lays great stress on these details. He writes so simply and yet fascinatingly that he entertains while he instructs. We recommend this book to the laity, as every intelligent person may read it with profit.

---

**Surgery:** Its Principles and Practice. In five volumes. By 66 eminent surgeons. Edited by **W. W. Keen,** M.D., LL.D., Hon. F.R.C.S., Eng. and Edin., Emeritus Professor of the Principles of Surgery and of Clinical Surgery, Jefferson Medical College, Phila. Volume IV. Octavo of 1194 pages, with 560 text-illustrations and 22 colored plates. Philadelphia and London: W. B. Saunders Company, 1908. (Per volume: cloth, $7.00 net; half morocco, $8.00 net.)

The fourth volume of Keen's Surgery, now under consideration, contains much interesting material as will be observed. Hernia, by William B. Coley; surgery of the rectum and anus, by Robert Abbe; examination of the urine in reference to surgical measures, by David L. Edsall; surgery of the kidney, the ureter, and the suprarenal gland, by Joseph Ranshoff; surgery of the bladder, by Brandsford Lewis; stone in the bladder, by Arthur Tracy Cabot; surgery of the prostate, by Hugh H. Young; surgery of the penis and urethra, by Orville Horwitz; surgery of the scrotum, testicle, spermatic cord, and seminal vesicles, by Arthur Dean Bevan; surgery of the intestines, but excluding the appendix, the rectum and the anus. Surgery of the omentum and mesentery, by Weller Van Hook and Allen D. Kanavel; surgery of the appendix vermiformis, by John B. Murphy; surgery of the ear, by Edward Bradford Dench; surgery of the eye, by George E. de Schwinitz; military surgery, by Surgeon General Robert M. O'Reilly; naval surgery, by Surgeon General P. M. Rixey; tropical surgery, by Walter D. McCaw; the influence of race, sex, and age on surgical affections, by William L. Rodman.

This group of subjects by these authors should be convincing proof of the great value of the book as a contribution to American surgery,—great surgeon, great book. Perhaps it were well if we said, great surgeons, great book, since so many of the contributors are, indeed, great surgeons.

This volume is one of the most valuable, if not the most valuable, of the series thus far issued. For example, it gives hernia in its latest operative phases, the prostate, the bladder, stone, the penis, the scrotum and testicle, the rectum an anus, all in most approved and latest surgery,—all being subjects that interest the general practitioner as well as the general surgeon. The mechanical execution and the art work of the book have never been excelled.

**Intestinal Autointoxication,** by **A. Combe,** M.D., professor of clinical pediatry at the University of Lausanne (Switzerland), together with an appendix on the lactic ferments with particular reference to their application to intestinal therapeutics, by **Albert Fournier,** formerly demonstrator at la Sorbonne, Paris. Only authorised English adaptation by **William Gaynor States,** M.D., clinical assistant in rectal and intestinal diseases at the New York Polyclinic. Eighteen figures, four colored. Rebman Company, New York. 1908. (Price, cloth $4.00.)

This subject has but lately, that is, comparatively recently received the study it merits. Many symptoms, we had almost said many diseases of the digestive tract, heretofore obscure or not explained, are made clear by an understanding of the processes of autointoxication. It seems to us the better term to apply to the condition is digestive autointoxication, the latter to be written as one word. And this brings us to say that the compounding of words in general should be very much reduced in these days of linotypes.

This may be termed a "personal" book, the author having studied the subject of gastrointestinal autointoxication, and having created for himself "personal methods and special procedures." Combe lays great stress on regimen. He considers it so important, that he has organised in many hotels throughout Switzerland tables of regimen where, under the direction of special and responsible cooks, his dietetic prescriptions are scrupulously followed!

While the general practitioner with a trend toward the investigation of gastrointestinal diseases may derive great benefit from a careful study of this book, it is of particular interest to the specialist in diseases of the digestive tract. Combe allots over 200 pages to the consideration of treatment, from which many valuable hints may be gleaned, but permeating through the text everywhere regimen is the shibboleth. The author and the translator are enthusiasts and have presented views that may well receive the attention of the American medical profession.

---

**American Practice of Surgery.** A complete System of the Science and Art of Surgery by representative surgeons of the United States and Canada. Edited by **Joseph D. Bryant,** M.D., and **Albert H. Buck,** M.D., New York. Complete in eight volumes. Vol. V. Imperial octavo, pp. 1010. Profusely illustrated. New York: William Wood & Company. 1909. (Price, cloth, $7.00.)

The fifth volume of this superb work is set apart to regional surgery. The contributors are, Edward Archibald, Montreal; Charles H. Frazier, Philadelphia; George C. Harlan, Philadelphia; Frank Hartly, New York; Charles H. Knight, New York; Robert Lewis, Jr., New York; Charles B. G. de Nancrède, Ann Arbor; James E. Newcombe, New York; Henry O. Reik, Baltimore; John D. Richards, New York; and James S. Stone, Boston.

The subjects are, surgical affections and wounds of the head; surgery of the cranial nerves; surgical diseases, certain abnormalities, and wounds of the face; hare lip and cleft palate; surgical diseases and wounds of the ear; sinus thrombosis of otitic origin, and suppurative diseases of the labyrinth; pyogenic diseases of the brain of otitic origin; surgical diseases and wounds of the pharynx; surgical diseases and wounds of the larynx and trachea; and laryngectomy.

From the foregoing it will appear that the volume is filled with material of particular interest to the specialist; though it must be understood that it is also full of interest to anybody who practises surgery in general. In other words we regard the volume as of great practical importance, one that is abreast of surgical science and art. It is a fitting companion to those which have gone before, and reflects credit upon the authors and editors, not to mention the publishers; for they should not be forgotten in an enterprise involving such a large outlay of money.

---

**Obstetrics for Nurses.** By **Joseph B. DeLee**, M.D., Professor of Obstetrics in the Northwestern University Medical School, Chicago. Third Revised Edition. 12mo, of 512 pages, fully illustrated. Philadelphia and London: W. B. Saunders Company. 1908. (Cloth, $2.50 net.)

From the first this book became most popular and with each succeeding edition its popularity has kept on increasing. It is a book, though intended in particular for nurses, that medical students may study with benefit.

Few changes were required in this edition, the work having reached a degree of perfection that was most remarkable. Most of the engravings are original and of excellent quality. It is a book that should be found in the hands of every nurse, even though she may not cultivate obstetric nursing.

---

**Treatment of the Diseases of Children.** By **Charles Gilmore Kerley**, M.D., Professor of Diseases of Children in the New York Polyclinic Medical School and Hospital. Octavo, pp. 597. Illustrated. Philadelphia and London: 1907. W. B. Saunders Co. (Price, $5.00.)

Again, we find the literature of diseases of children enriched by a contribution of no mean proportions, and this whether we consider the size of the volume before us, or the material which it contains. Kerley is a teacher of distinction and one who has mastered his subject. His work at the polyclinic has been of a nature to give him an enviable standing throughout the country, and it has served to familiarise him with the needs of the general practitioner regarding the diseases of infants and young children.

The treatise differs from the ordinary work on this subject because of the fact that it does not stop with the treatment of disease by drugs or with the stereotype directions for infant feeding. It takes up the defective nutrition and development of advancing childhood and instructs as to the management by muscular gymnastics and other important methods which tend to promote symmetry of growth.

The illustrations, especially those showing the treatment of scoliosis, are of the first order. The only criticism relating to the pictorial part of the book we would make, is that they are too few in number. Pictures play such a large part in medical instruction at the present time, it should be the aim of every author to make elaborate illustrations of his subject whenever and wherever engravings can be made use of with appropriateness. We commend this work to every general practitioner who assumes in any degree the treatment of the diseases of children.

---

**International Clinics**. A Quarterly of Illustrated Clinical Lectures and especially prepared articles on Treatment, Medicine, Surgery, Neurology, Pediatrics, Obstetrics, Gynecology, Orthopedics, Pathology, Dermatology, Ophthalmology, Otology, Rhinology, Laryngology, Hygiene and other topics of interest to students and practitioners. Edited by **W. T. Longcope**, M.D., Volume IV., eighteenth series. Philadelphia and London: J. B. Lippincott Co. 1908. (Cloth, $2.00.)

This volume contains four articles on treatment; four on medicine; five on surgery; three on obstetrics and gynecology; one on hygiene; two on neurology; one on laryngology; and two on pathology.

Among the important articles are Lichty's on the treatment of gastric ulcer; Wainwright's on gout; Weber's on splenomegaly; Nicholls's on acute dilatation of the stomach; Vaughan's on fractures of the skull; McGregor's on traumatic lesions of the semilunar cartilages of the knee joint; Ely's on Bier's hyperemia; Cumston's on paronychia lateralis; Brown's on the rôle of insects in the transmission of disease; Scarlett's on the relation which hypertrophy of the various tonsils of Waldeyer's ring bears to the etiology of disease; Dunn's on the serum treatment of epidemic cerebrospinal meningitis; and Deaderick's on the etiology and treatment of pernicious malaria.

There are several other excellent articles of lesser importance which we cannot now mention by title. The illustrations while not profuse are mainly of good quality, giving emphasis to the text. This book as a whole is of excellent quality and will prove of usefulness to the general practitioner.

---

**The Baby. Its Care and Development.** For the Use of Mothers. By **Le Grand Kerr,** M.D., Author of Diagnostics of the Diseases of Children. 12mo, pp. 150. Illustrated. Brooklyn: Albert T. Huntington. 1908. (Cloth, $1.00.)

The baby is the most important member of every young household; it always occasions much anxiety even when in good health. Kerr has told, in concise manner some important things that every young mother should know. The book is small but the table of contents is large. The illustrations serve to draw attention to many salient features that otherwise might escape close attention. The author understands his subject, and is not verbose in dealing with it. We think it would be well for every young mother, as well as every prospective mother to obtain and keep "The Baby" at hand for reference.

---

**Textbook of General Bacteriology.** By **Edwin O. Jordan,** Ph.D. Professor of Bacteriology in the University of Chicago and in Rush Medical College. Fully illustrated. Philadelphia and London: W. B. Saunders Company. 1908. (Price, $3.00 net.)

A knowledge of bacteriology becomes, of necessity, a part of the accomplishment of the medical student at the present time; at least he should acquire a working knowledge, so to speak, of the general subject of bacteriology. This author is a conscientious instructor and his book appeals to the searcher for this knowledge, increasingly so with every page one reads.

Jordan does not claim to present an exhaustive work embracing all sides of bacteriology; but rather to give the fundamental principles of the subject, together with the methods of laboratory work. The author lays great stress upon the importance of thorough training, and refers those who wish to pursue the subject further to larger treatises. It is one of the best practical works on bacteriology we have examined, the classification of bacteria, especially being excellent. The illustrations, too, deserve mention, as being of the better class, admirably fulfilling their purpose.

---

**Therapeutics of the Circulation.** Eight lectures delivered in the Spring of 1905, at the University of London. By **Lauder Brunton,** Consulting Physician to St. Bartholomew's Hospital. Small 8vo. pp. 280. Illustrated. Philadelphia: P. Blakiston's Son & Co. ($1.50.)

Lauder Brunton is a name that commands respect wherever medicine is known and taught. He has condensed into these five lectures a vast amount of valuable information on a very important topic, embracing some of the more recent work in the physiological laboratory at the University of London. These lectures were delivered in accordance with the general purpose of the university establishing the laboratory—namely, to present the results of recent investigations by the investigators themselves.

This is one of the most interesting, not to say instructive, books we have read in a long time. It is a subject that every physician should become familiar with. Every now and then we hear of the death at a banquet of a prominent speaker which we know is due to defects of circulation; also, that a speaker was seized with "acute indigestion," which is usually due to defective circulation, perhaps arteriosclerosis. This book will serve to explain the causes of such attacks. The increase of bloodpressure due to nerve strain, in a person with rigid or hardened arteries, is a fruitful source of sudden prostration and perhaps death.

Brunton points out the means of prolonging life even in the presence of grave circulatory defects. He should have a large audience, and his book is the medium through which he will obtain one.

---

**Obstetrics and Gynecologic Nursing.** By **Edward P. Davis,** A.M. M.D., Professor of Obstetrics in the Jefferson Medical College, Philadelphia; Obstetrician and Gynecologist to the Philadelphia Hospital; Consultant to the Preston Retreat. Third edition, thoroughly revised. Philadelphia and London: W. B. Saunders Company. 1908. (Price, $1.75 net.)

The author of this book is an experienced teacher of obstetrics and knows just the kind of instruction of which nurses stand most in need. If a nurse carefully heeds the instruction given by Davis she will be competent to manage a labor in an emergency without a physician. Indeed, every obstetric nurse should be prepared for such a contingency. The labor may be unusually rapid, or the doctor delayed in arriving,—these and several other circumstances may serve to thrust responsibility upon the nurse, and she must be equal to the occasion.

Davis has revised and enlarged this edition, making it one of the most complete works of its kind yet issued. It deals with gynecologic as well as obstetric nursing, and may be regarded as a textbook for nurses, just as the larger works on medicine and surgery are called textbooks, when compared with handbooks or manuals. No mistake can be made by the nurse who purchases this book.

---

**Textbook of Diseases of Women.** By **Charles B. Penrose,** M.D., Ph.D., formerly Professor of Gynecology in the University of Pennsylvania; Surgeon to the Gynecean Hospital Philadelphia. With 225 illustrations. Sixth edition revised. Philadelphia and London: W. B. Saunders Company. 1908. (Price, cloth, $3.75; half-morocco, $5.25 net.)

This book, though primarily intended for the medical student is full of material of value to the general practitioner. It is written in plain language and is concise in its statements. In most instances but one plan of treatment is recommended, thus avoiding that confusion resultant from describing the methods of different authors or teachers. The illustrations, many being drawn from nature, elaborate the text with intelligence. The first edition was put forth in 1897, now comes the sixth eleven years afterward, showing the popularity of the work. This edition has been thoroughly revised, bringing it forward to the immediate present in the essentials required by modern gynecology.

**Applied Surgical Anatomy,** Regionally Presented. By **George Woolsey,** A.B., M.D., Professor of Anatomy and Clinical Surgery in Cornell University Medical College, New York. Second edition. Octavo volume of 601 pages, with 200 illustrations in black and colors. Lea & Febiger, Philadelphia and New York, 1908. (Cloth, $4.50, net.)

Anatomy and surgery go hand in hand, and have done so since Harvey's discovery of the circulation of the blood. Anatomy forms the base of the medical sciences and must be acquired in particular by him who would become a surgeon. It is well, too, for the prospective surgeon to teach anatomy for a decade, before turning his entire attention to surgery. Professor Woolsey has taught anatomy for eighteen years, and, besides, is a practical surgeon. He is fitted therefore, in a special degree, to present such a work as this.

This edition has been thoroughly revised resulting in much enlargement, as well as an increase in the number of illustrations. The use of colors adds much to the value of pictures relating to anatomy or surgery, and in this book discretion has been shown in this regard. It is a work well adapted to the general practitioner who must needs do surgery, as well as to the specialist in the field of surgery.

---

**Vaccine Therapy and the Opsonic Method of Treatment.** A short compendium for general practitioners, students and others. By **R. W. Allen,** M.D., B.S. (Lond.) late pathologist to the Royal Eye Hospital, etc. Second edition. Octavo, pp. 244. Philadelphia, Pa. Blakiston's Son & Company. 1908. (Price, $2.00, net.)

So much is being said and written of opsonins, and of vaccine therapy, that such a manual as this is not only timely, but even a necessity. The progress even in a year's time has been remarkable and no one can predict what another year will unfold in this method of treatment. Of one thing, however, we are quite certain, in order to be successful it must be administered by skilful hands. This monograph, written by a man of experience, will do much to develop a proper understanding of the subject among general practitioners and students, as well as others who may be especially interested. The first edition of this book was sent forth in November, 1907, and in less than a year a new edition was needed. The author has corrected some defects and slight errors that appeared in the first book, while so much enlargement became necessary, that this may be considered almost a new monograph. At all events it will find its way undoubtedly into the hands of every person interested in this line of study.

---

**Gonorrhea in Women.** By **Palmer Findley,** M.D., Professor of Gynecology in the College of Medicine of the University of Nebraska, Omaha. Pp. 112. St. Louis: C. V. Mosby Co. (Cloth, $2.00.)

A monograph on this subject thirty years ago would have astonished the professional world; now, it is almost remarkable that it should not have been written sooner. Noeggerath, as early as 1878 published his wonderful declaration that 80 per cent. of married men have had gonorrhea; that 90 per cent. of these have never been cured, and that of five married women, three have gonorrhea. His dictum was based on observations made in the city of New York. Scarcely had Noeggerath published his observations when (1879) Neisser announced his discovery of the gonococcus, which revolutionised the pathology and treatment of gonorrhea. Findley discusses with intelligence the relationship of latent gonorrhea to disease of the tubes, ovaries, and pelvic tissues in general, including the peritoneum. He gives an extensive bibliography mostly from the German. There are no illustrations, though the book is printed on heavily calendered paper. It is an admirable essay.

---

**Practical Points in Anesthesia.** By **Frederick-Emil Neef,** New York. Small 12 mo, pp. 51. New York: Surgery Publishing Co.

A primer of the size of this one is an excellent reference book for the anesthetist to carry in his pocket. Among the topics dealt with are induction of anesthesia, cardiac and respiratory collapse, when shall the patient be declared ready for operation, maintenance of the surgical plane of anesthesia, important reflexes, vomiting during anesthesia, obstructed breathing, use of the breathing tube, indications for stimulation, influence of morphine on narcosis, general course of anesthesia, awakening, recession of tongue after narcosis, postoperative distress, minor anesthesia with ethyl chloride, intubation anesthesia, etc., etc.

---

**The Campaign against Tuberculosis in the United States,** including directory of institutions dealing with tuberculosis in the United States and Canada. Compiled under the direction of the National Association for the Study and Prevention of Tuberculosis by **Philip P. Jacobs,** New York: Charities Publication Committee 1908. (Price, $1.00 net.)

This remarkable book is published by the charities publication committee of the City of New York, the funds for which, however, were supplied by the Russell Sage Foundation. It is compiled under the direction of the National Association for the Study and Prevention of Tuberculosis by Philip P. Jacobs. The book is in reality a directory of the sanatoria, hospitals, and day camps for the treatment of tuberculosis in the United States and Canada. It gives, also, hospitals for the insane that make special provision for tuberculosis inmates, as well as penal institutions that make similar provisions. It also gives dispensaries and clinics for the special treatment of tuberculosis patients. Finally, it gives a summary of the legislation enacted in the several states and territories relating to tuberculosis. It is handsomely printed and bound and is sold at cost, the price being one dollar.

---

**A Handbook of Suggestive Therapeutics, Applied Hypnotism, Psychic Science.** By **Henry S. Munro,** M.D., Americus, Ga. 12 mo, pp. 360. Second edition. St. Louis: C. V. Mosby Co. (Cloth, $3.00.)

The prefaces to this work not being dated, and as we did not see the first edition we accept the statement of the author, that the first edition was a large one and was exhausted nine months after its appearance. This is abundant evidence that the book was acceptable to the profession, that is,, that portion which employs this method of treatment. The volume is full of examples of the successful application of suggestion and hypnotism. It will prove interesting to any physician of experience who has the time to read it, but we doubt the propriety of placing such a work in the hands of the young and inexperienced practitioner. The pages are marred by the profuse employment of heavy faced type, and errors of grammar occur occasionally, but in general it represents its class.

---

**Cataract Extraction.** By **H. Herbert,** F.R.C.S., late Lieutenant Colonel I. M. S., Professor of Ophthalmic Medicine and Surgery in Grant Medical College, and in charge of the Sir Cowasjee Jehangir Ophthalmic Hospital, Bombay. Octavo, pp. 391. New York: William Wood & Company. 1908.

The extraction of cataract has been considered one of the more delicate surgical procedures, and ophthalmic surgeons, especially the juniors, will enjoy a book on the subject by so experienced an operator as Herbert. We do not mean to be understood that even the older ophthalmologists may not derive benefit from the reading or study of the book, at least in some of its parts. The author bases his monograph on an experience of 5,000 cataract operations, which he is modest enough to say is comparatively a small number for an ophthalmic surgeon in India, adding that in busy seasons, they rarely see their cases after operation, for lack of time.

Herbert describes the operation in minute detail, devoting a long chapter, 136 pages, to this purpose. In another long chapter, 100 pages, he presents variations to the ordinary procedure and their value. After complications receive liberal attention in generous space. The entire text is written in excellent English, simple, concise, instructive.

---

**Arteriosclerosis.** By **Louis M. Warfield,** M.D., Instructor in Medicine in Washington University, Medical Department. 12 mo, pp. 165. Illustrated. St. Louis: C. V. Mosby Co. (Cloth, $2.00.)

It is fitting in these days of hurry and worry, of overwork and under rest, that such a book as this should be offered to the professional public. It is quite true that arteriosclerosis is a product of present day life, of high living and rush. Degenerative changes take place readily in the midst of twentieth century civilisation; and especially in the arteries with the increased blood-pressure incident to the excitement, the rapidity of movement, the strain of every day life, whether of business or pleasure. The cardiovascular system is sensitive and must be taken care of during early and middle life to permit of a comfortable old age.

Arteriosclerosis is a disease of prevention rather than cure. While it is usually a disease of advanced life yet sometimes children are affected with it. Outdoor exercise is the chief remedy—but the book must be read to be appreciated. It is an excellent monograph on a most important topic and every physician in active practice, whether generalist or specialist, would do well to familiarise himself with its pages. We could wish the author had ended fewer of his sentences with "etc"—indeed, this abbreviation should never end a sentence,—yet, as a whole, the book contains few errors that mar its handsome pages.

